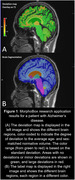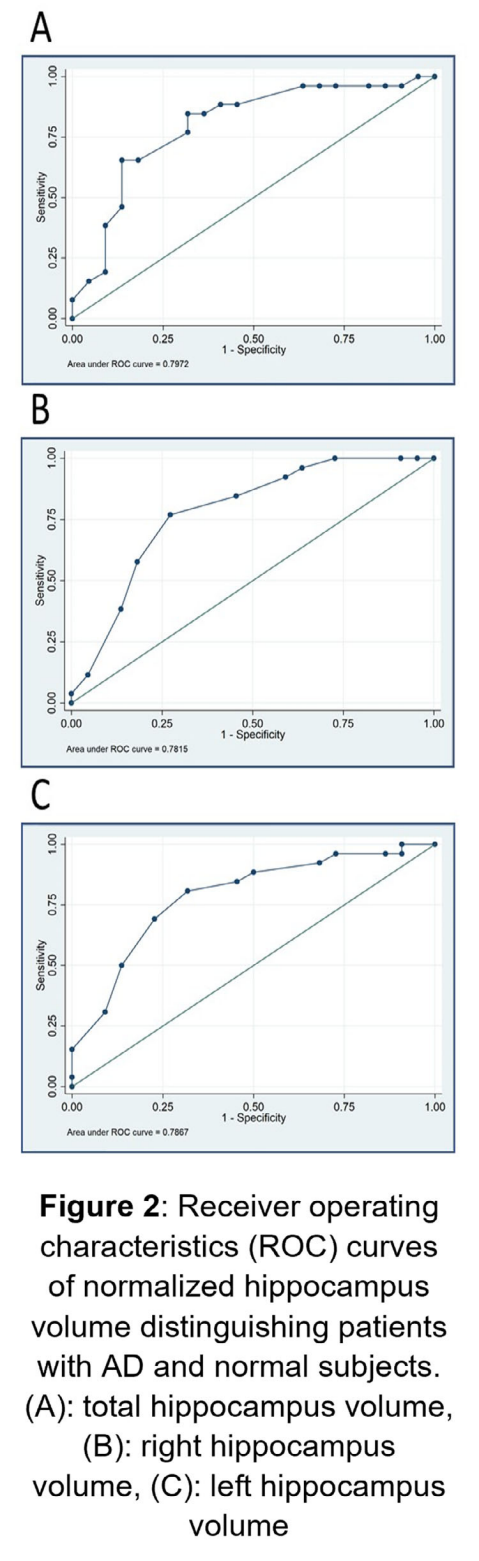# Hippocampal Volumetry: A Promising and Accessible Tool for Early AD Diagnosis in Regions with Limited 18F‐FDG PET Imaging Access?

**DOI:** 10.1002/alz70856_102176

**Published:** 2025-12-24

**Authors:** Bénédicte Maréchal, Tommaso Di Noto, Thanchanok Jomsak, Attapon Jantarato, Dheeratama Siripongsatian, Chanisa Chotipanich, Attapon Jantarato

**Affiliations:** ^1^ Swiss Innovation Hub, Siemens Healthineers International AG, Lausanne, Switzerland; ^2^ LTS5. École Polytechnique Fédérale de Lausanne, Lausanne, Switzerland; ^3^ Lausanne University Hospital, Lausanne, Switzerland; ^4^ National Cyclotron and PET Centre, Chulabhorn Hospital, Chulabhorn Royal Academy, Bangkok, Thailand

## Abstract

**Background:**

Hippocampal atrophy and hypometabolism are key neuroimaging biomarkers for early diagnosis of Alzheimer's Disease (AD). Artificial intelligence methods for quantitative automated volumetric analysis of brain magnetic resonance imaging (MRI) are enabling objective assessment of brain atrophy in a fast and accessible way. Thus, the objective of this work is to compare hippocampal volume derived from MRI with the hypometabolism observed in 18F‐FDG positron emission tomography (PET) in the context of early diagnosis of AD.

**Methods:**

Twenty‐two patients with AD (11 women, [51–84yo], mean age±SD: 69.91±9.51) and twenty‐six normal individuals (16 women, age range [51–80yo], mean age±SD: 66.23±6.87) were enrolled in this study. Structural T1‐weighted and 18F‐FDG scans were acquired on a 3T MR‐PET system (Biograph mMR, Siemens Healthineers, Forchheim, Germany). A deep‐learning‐based research application (MorphoBox) was used to derive brain volume normalized by the total intracranial volume from MRI (Figure 1), and *p*‐mod software was applied to calculate standardized uptake value ratio (SUVR) from PET imaging. Pearson correlations between normalized brain volumes and SUVR, as well as visual interpretations from diagnostic radiologists, were assessed. Receiver operating characteristics (ROC) analyses were conducted to investigate the discrimination ability of hippocampus relative volume on one hand and SUVR on the other hand.

**Results:**

A significant difference in SUVR normalized by cerebellar SUV was observed between the AD and control groups in the following brain regions: hippocampus (*p* <0.001), frontal lobe (*p* <0.001), parietal lobe (*p* <0.001), temporal lobe (*p* <0.001), occipital lobe (*p* = 0.001), cingulate (*p* <0.001), caudate (*p* = 0.002) and thalamus (*p* <0.001). There was a strong correlation between SUVR and normalized volume, particularly in hippocampus, frontal, parietal, and temporal regions (ρ=0.60, 0.64, 0.67, and 0.73, respectively). The ROC curve for hippocampal relative volume yielded an AUC of 0.79 indicating good discrimination abilities (Figure 2).

**Conclusion:**

Our study demonstrates a strong correlation between hippocampal volumetry from MRI and FDG PET brain imaging. The MorphoBox research application under study could serve as a valuable screening tool in centers with restricted access to PET imaging. Additionally, our findings indicate that hippocampal volumetry exhibits superior discrimination power in the sample under study. We recommend further evaluation with a larger sample size in future research.